# R-(+)-propranolol for infantile hemangioma: *β*-adrenoceptor-independent mechanisms and clinical translation prospects

**DOI:** 10.3389/fmed.2026.1863768

**Published:** 2026-07-30

**Authors:** Wei Peng, Feng Chen, Yuyang Zheng, Yong Zeng, Zhiqi Zhao, Qian Liu, Haijin Liu

**Affiliations:** 1 Department of Pediatric Surgery, First Affiliated Hospital of Gannan Medical University, Ganzhou, China; 2 Department of Clinical Research Center of Vascular Abnormalities of Jiangxi Province, Ganzhou, China; 3 The First Clinical Medical College of Gannan Medical University, Ganzhou, China; 4 Jiangxi Provincial Key Laboratory of Traditional Chinese Medicine Prevention and Treatment of Hemangioma, Jiangxi University of Chinese Medicine, Nanchang, China

**Keywords:** angiogenesis, chiral drug, infantile hemangioma, R-(+)-propranolol, SOX18, ***β***-adrenoceptor-independent mechanism

## Abstract

Infantile hemangioma (IH) is the most prevalent benign vascular tumor in infancy, and propranolol is the first-line standard pharmacotherapy for high-risk IH. The clinically used formulation of propranolol is an equimolar 1:1 racemic mixture of two enantiomers, S-(−)-propranolol and R-(+)-propranolol. The traditional mechanistic hypothesis is centered on the *β*-adrenoceptor blockade pathway mediated by the S-enantiomer, yet this framework fails to fully explain the dose–response relationship and long-term involution effect of propranolol in IH treatment. Recent studies have revealed that R-(+)-propranolol, which exhibits negligible *β*-adrenoceptor blocking activity, exerts a remarkable and independent anti-IH effect, indicating the existence of a core *β*-adrenoceptor-independent mechanism of action. This review systematically integrates the preclinical evidence for the β-independent anti-IH activity of R-(+)-propranolol, elucidates the discovery logic of SOX18 as the core molecular target, identifies key translational gaps (zero clinical trials, lack of infant enantiomer-specific PK data, chiral formulation challenges), and proposes a dual-track model of propranolol action in IH. The authors further clarify the critical gaps in clinical translation in this field and provide a theoretical basis for the precision therapy of IH and the development of novel chiral pharmaceuticals.

## 1 Introduction

Infantile hemangioma (IH) is the most common benign vascular tumor in infancy, arising from the abnormal proliferation of vascular endothelial cells (1, 2). Its natural clinical course is classically divided into three sequential phases: the proliferative phase, the stable phase, and the involution phase. Approximately 10 to 15% of cases are classified as high-risk IH, which includes lesions located in critical anatomical sites (such as the periocular region, airway, and liver), as well as large segmental or giant proliferative lesions. These high-risk lesions can lead to permanent disfigurement, organ dysfunction, and even life-threatening events (3–5), in addition to imposing a significant psychological burden on affected families (3), thus necessitating active clinical intervention (4–6). Since the landmark discovery of the therapeutic efficacy of propranolol against IH in 2008, it has rapidly replaced traditional therapeutic regimens including systemic corticosteroids, and has become the first-line standard treatment for high-risk IH, with its efficacy and safety well validated by numerous high-quality clinical studies (7, 8).

In clinical pharmacology, the differential pharmacodynamic and pharmacokinetic (PK) profiles of enantiomers within a racemic drug are frequently overlooked, often leading to suboptimal therapeutic precision. Clinically used propranolol is a non-selective *β*-adrenoceptor blocker, formulated as a racemic mixture of S-(−)-propranolol and R-(+)-propranolol (9). Among the two enantiomers, the β-adrenoceptor blocking potency of the S-enantiomer is approximately 100-fold higher than that of the R-enantiomer, and it has been historically recognized as the primary active component of the racemic formulation (10, 11). Based on its *β*-adrenoceptor blocking activity, the prevailing mechanistic hypothesis posits that propranolol exerts its therapeutic effects via three core pathways: vasoconstriction, inhibition of angiogenesis, and induction of endothelial cell apoptosis (6, 12, 13). However, this theory fails to explain a number of clinical and experimental observations, First, at effective clinical doses, measured propranolol plasma concentrations (approximately 0.1–1.5 μM) are about 100-fold higher than those required for complete *β*-adrenoceptor saturation (approximately 1–10 nM) (14). Second, whether highly selective *β*-adrenoceptor blockers are inferior to racemic propranolol remains controversial: some studies have shown non-inferiority of the selective β₁-blocker atenolol (15). These discrepancies strongly suggest the existence of a *β*-adrenoceptor-independent mechanism underlying the therapeutic effects of propranolol.

Recent groundbreaking studies have revealed that R-(+)-propranolol, which exhibits negligible *β*-adrenoceptor blocking activity, exerts anti-IH activity comparable to or even superior to that of the racemic mixture in both *in vitro* and *in vivo* models, making it the key breakthrough for deciphering the *β*-adrenoceptor-independent mechanism of propranolol (11, 16).

Although studies on R-(+)-propranolol are still limited, accumulating evidence has revealed a *β*-adrenoceptor-independent mechanism that differs from the traditional β-blockade paradigm. The field currently faces several key knowledge gaps: (i) the β-independent anti-IH mechanism of R-(+)-propranolol has not been systematically integrated; (ii) evidence for SOX18 as the core molecular target remains fragmented; (iii) specific translational barriers exist (zero clinical trials, lack of infant enantiomer-specific PK data, chiral formulation challenges); and (iv) the traditional mechanism fails to explain the observed dose–response relationships and enantiomer differences. To address these gaps, this review systematically integrates all preclinical evidence for the *β*-independent anti-IH activity of R-(+)-propranolol, elucidates the SOX18-centered molecular mechanism with a stepwise logical framework from transcriptomics to functional validation, identifies specific translational gaps to guide future research, and proposes a dual-track model that reconciles clinical observations with enantiomer-specific actions, thereby revising the traditional single-*β*-blockade hypothesis.

## 2 Core evidence for the β-adrenoceptor-independent anti-IH activity of R-(+)-propranolol

The extremely low β-adrenoceptor affinity of R-(+)-propranolol makes it an ideal tool to distinguish whether the therapeutic efficacy of propranolol is dependent on β-adrenoceptor blockade. Accumulating *in vitro* and *in vivo* studies have provided robust evidence for its independent anti-IH activity.

### 2.1 Direct activity validation in *in vitro* cell models

Multiple studies have supported by preclinical evidence that R-(+)-propranolol directly regulates the biological behaviors of core cellular components in IH in a *β*-adrenoceptor-independent manner. In a human-derived hemangioma endothelial cell model, R-(+)-propranolol significantly downregulated the expression of angiopoietin-like protein 4 (ANGPTL4), a key molecule involved in angiogenesis, with this effect significantly superior to that of S-(−)-propranolol. Meanwhile, R-(+)-propranolol directly inhibited the differentiation of vascular endothelial cells, an effect not observed with S-(−)-propranolol (11).

Hemangioma-derived stem cells (HemSCs) are the cellular origin of tumorigenesis, proliferation, and recurrence of IH, as well as the core therapeutic target of propranolol. Studies have suggested that the inhibitory effect of propranolol on HemSCs is independent of *β*-adrenoceptor blockade: R-(+)-propranolol treatment significantly altered the transcriptomic profile of HemSCs and inhibited their differentiation toward vascular endothelial cells (11, 17). Moreover, R-(+)-propranolol also promotes adipogenic differentiation and lipid accumulation in HemSCs (18, 19). This effect is completely inconsistent with its negligible *β*-adrenoceptor affinity, directly supporting the existence of its independent pharmacological activity (19, 20).

### 2.2 Efficacy validation in *in vivo* animal models

The findings from *in vitro* studies have been further validated in *in vivo* models. In a bEnd.3 hemangioma tumor-bearing mouse model (the bEnd.3 cell line is a murine brain endothelioma cell line immortalized by retroviral expression of polyoma middle T antigen), intervention with R-(+)-propranolol significantly inhibited tumor growth, while no significant difference was observed between the S-(−)-propranolol group and the placebo group (20). This directly was supported by preclinical evidence that the anti-IH effect of propranolol in the *in vivo* physiological environment is mainly mediated by the R-enantiomer, independent of *β*-adrenoceptor blockade (21). Furthermore, using a human primary HemSC xenograft model, Seebauer et al. also provided preclinical evidence that R-(+)-propranolol significantly inhibits vessel formation *in vivo* (11). Although current data on R-(+)-propranolol monotherapy in patient-derived human IH xenograft models remain limited, the available core animal experiments have provided the most direct *in vivo* evidence for the *β*-adrenoceptor-independent mechanism (21).

### 2.3 Reappraisal of the traditional β-adrenoceptor-dependent mechanism

The independent activity of R-(+)-propranolol does not completely negate the *β*-adrenoceptor-dependent pathway, but rather reshapes the cognitive framework of the mechanism of action of propranolol. The β-adrenoceptor blockade mediated by the S-enantiomer explains the early hemodynamic changes after drug administration, including rapid fading of lesion color and softening of tumor texture, serving as the “rapid auxiliary pathway” of treatment. However, the core effect that may predominantly contribute to the long-term, substantial involution of the lesion is mainly mediated by the *β*-adrenoceptor-independent pathway of the R-enantiomer. This model provides a plausible explanation for the clinical phenomenon of the wide therapeutic window of propranolol: the plasma concentration achieved by clinical administration is far higher than that required for β-adrenoceptor saturation, and the improved therapeutic efficacy brought by dose escalation is derived from the dose-dependent effect of the R-enantiomer on non-receptor targets.

Table 1 summarizes the key preclinical studies on R-(+)-propranolol in infantile hemangioma models, including model type, cell source, major findings, S-enantiomer control, dosage, and limitations.

**Table 1:** Summarizes the key preclinical studies on R-(+)-propranolol in infantile hemangioma models.

Study	Model type	Cell/animal source	Key findings with R-(+)-propranolol	S-(−)-propranolol control	Dosage/concentration	Major limitations
Seebauer, 2022 (11)	*In vitro* + xenograft	Human HemSCs (patient-derived)	Inhibited HemSC-to-endothelial differentiation; reduced vessel formation *in vivo*; R-atenolol equally effective	Tested, weaker inhibition than R-	10 μM *in vitro*; 5–12.5 mg/kg (i.p., b.i.d.)	No direct measurement of SOX18-DNA binding *in vivo*
Sasaki, 2019 (20)	*In vitro* + tumor-bearing mouse	bEnd.3 (murine endothelioma cell line)	Inhibited tumor growth; downregulated ANGPTL4	Also downregulated ANGPTL4	2 mg/kg (i.p., b.i.d.)	Non-human, non-IH-specific model; immortalized cell line
Overman, 2019 (16)	*In vitro* binding + reporter assay	HEK293 cells	Directly bound SOX18 HMG domain; suppressed transcriptional activity	No binding or inhibition	10–50 μM	No *in vivo* efficacy data; only biochemical/cell-based
Holm, 2025 (17)	*In vitro* + xenograft	Human HemSCs	Blocked SOX18-mevalonate pathway axis; simvastatin phenocopied effect	Not tested	10 μM *in vitro*; 10 mg/kg (i.p., b.i.d.)	No S-enantiomer control; human equivalent dose unclear
Chen, 2026 (27)	*In vitro*	Human HemSCs	Promoted adipogenesis and subsequent apoptosis (PERK pathway)	Not tested	40 μM	No endothelial differentiation endpoint; high concentration
Tan, 2025 (18)	*In vitro*	Human HemSCs	Decreased lipid accumulation	Not tested	20 μM	Mechanism not fully elucidated; no *in vivo* data

HemSCs, hemangioma-derived stem cells; HMG, high mobility group; PERK, protein kinase RNA-like endoplasmic reticulum kinase; i.p., intraperitoneal; b.i.d., twice daily; IH, infantile hemangioma. For Sasaki et al. (20), the bEnd.3 cell line is a murine endothelioma cell line immortalized by retroviral expression of polyoma middle T antigen, which does not fully recapitulate human IH pathophysiology.

Data in this table are compiled from Refs. (11, 16, 17, 19, 20, 21).

## 3 Core molecular mechanisms underlying the anti-IH effects of R-(+)-propranolol

Accumulating studies have strongly identified the transcription factor SOX18 as the core molecular target mediating the β-adrenoceptor-independent effects of R-(+)-propranolol, with additional auxiliary regulatory pathways also implicated (16).

### 3.1 Core target: the transcription factor SOX18

Researchers first employed transcriptomics to identify downstream effectors of R-(+)-propranolol. Bulk RNA-Seq of R-(+)-propranolol-treated HemSCs revealed significant downregulation of mevalonate pathway (MVP) genes, a pathway known to be regulated by SOX18 (17). Subsequently, Overman et al. used biochemical, biophysical, and single-molecule imaging assays to demonstrate that R-(+)-propranolol directly binds the high-mobility group (HMG) domain of SOX18, disrupts its chromatin-binding dynamics, and reduces SOX18 homodimer and SOX18-RBPJ (recombination signal binding protein for immunoglobulin kappa J region) heterodimer formation, thereby suppressing SOX18 transcriptional activity (16). Functional validation further supports the essential role of SOX18: the specific SOX18 small-molecule inhibitor Sm4 recapitulates the anti-angiogenic effect of R-(+)-propranolol *in vivo* (11); and SOX18 knockdown in HemSCs abolishes the regulation of MVP gene expression by R-(+)-propranolol (17). These stepwise lines of evidence establish SOX18 as the core direct target through which R-(+)-propranolol exerts its anti-IH effects.

SOX18 is a member of the SRY-box (SOX) transcription factor family, serving as a core “master switch” for vascular and lymphatic development (22), and its aberrant activation is a key pathogenic mechanism driving the abnormal proliferation and differentiation of endothelial cells in IH (16). SOX18 is highly expressed in proliferative IH tissues, with its expression level positively correlated with the proliferative activity of the lesions, thus representing a key therapeutic target for IH (17).

Multiple pioneering studies have provided preclinical evidence that the inhibitory effect of propranolol on SOX18 transcriptional activity exhibits strict chiral selectivity. In direct assays of SOX18 transcriptional activity (e.g., reporter gene assays and chromatin binding dynamics), only R-(+)-propranolol suppresses SOX18-mediated transactivation, whereas S-(−)-propranolol has no such effect (16, 17). Of note, for the downstream molecule ANGPTL4, Sasaki et al. reported that S-(−)-propranolol also downregulates its expression (20), suggesting that the S-enantiomer may affect angiogenesis-related molecules through other pathways. However, it should be noted that the bEnd.3 cell line used by Sasaki et al. is a murine endothelioma cell line (immortalized by retroviral expression) with limited relevance to human IH; thus, this finding should be interpreted with caution.

By inhibiting SOX18, R-(+)-propranolol blocks the expression of its downstream angiogenesis-related target genes at the transcriptional source, thereby inhibiting the differentiation of HemSCs into endothelial cells, halting endothelial cell proliferation and angiogenesis, and achieving long-term suppression of IH growth. This constitutes the primary mechanism in preclinical studies underlying its *β*-adrenoceptor-independent effects. This effect has been supported by preclinical evidence to be comparable to that of specifically developed small-molecule SOX18 inhibitors, providing core theoretical support for the clinical translation of R-(+)-propranolol (11, 16).

### 3.2 Other auxiliary regulatory pathways

In addition to the core SOX18-mediated pathway, R-(+)-propranolol exerts synergistic anti-IH effects through other *β*-adrenoceptor-independent pathways. As described above, R-(+)-propranolol specifically downregulates the expression of ANGPTL4 in IH cells (20), and ANGPTL4 is a key secretory protein that regulates angiogenesis, indicating that this pathway further enhances its anti-angiogenic effects.

While racemic propranolol has been shown to modulate signaling pathways related to cell proliferation and migration (such as the PI3K/AKT/mTOR and RAC1/PAK1 axes), it is crucial to emphasize that the chiral selectivity of these regulatory effects remains completely unverified. Therefore, the direct contribution of R-(+)-propranolol to these auxiliary pathways should be interpreted with caution and warrants rigorous future investigation.

## 4 Gaps in clinical translation and future perspectives

### 4.1 Core limitations in the current field

Although the β-adrenoceptor-independent anti-IH activity of R-(+)-propranolol has been validated in preclinical models, a substantial disconnect remains between basic research and clinical practice. These critical gaps are concentrated in three main aspects.

First, there is a complete absence of clinical evidence. As of April 2026, no clinical trials of R-(+)-propranolol monotherapy for IH have been published, nor have head-to-head comparative studies with racemic propranolol. All available clinical data are derived from racemic formulations, which cannot distinguish the respective contributions of the R- and S-enantiomers to therapeutic efficacy and safety.

Second, there is a lack of pharmacokinetic (PK) data in the infant population. While stereoselective differences in the metabolism of propranolol enantiomers have been identified in adults: after oral administration of racemic propranolol, the peak plasma concentration and area under the concentration-time curve (AUC) of S-propranolol are significantly higher than those of R-propranolol (8, 10), the absorption, distribution, metabolism, and excretion (ADME) characteristics of the R- and S-enantiomers in infants remain completely unknown. Furthermore, the potential for *in vivo* metabolic interconversion between enantiomers (R-to-S) remains unexplored in this vulnerable population.

Third, significant pharmaceutical and formulation barriers must be addressed. Chiral purity is a critical quality attribute; any contamination with the S-enantiomer would reintroduce *β*-adrenoceptor-mediated adverse effects. Additionally, the development of an infant-appropriate oral formulation (e.g., taste-masked solution) with long-term chiral stability is required, as current racemic formulations are not optimized for R-monotherapy.

### 4.2 Clinical translation value and future research directions

The core advantage of developing R-(+)-propranolol monotherapy lies in the significant improvement in treatment safety. The clinical adverse events associated with racemic propranolol, including bradycardia, hypotension, bronchospasm, and hypoglycemia (23, 24), are all potentially attributed to the β-adrenoceptor blocking activity of the S-enantiomer. Particularly for developing infants, the potential impact of long-term β-adrenoceptor blockade on neurodevelopment remains a major clinical concern. Specifically, the elimination of S-enantiomer-related cardiopulmonary risks could potentially streamline clinical protocols, possibly reducing the necessity for rigorous pre-treatment cardiovascular screening and frequent intra-treatment vital sign monitoring, thereby lowering both the medical burden and parental anxiety.

Future research should focus on three core directions to promote the translation of basic discoveries into clinical practice. First, the initiation of pivotal clinical trials. Priority should be given to conducting multicenter, randomized, double-blind phase II clinical trials to compare the efficacy and safety of R-(+)-propranolol monotherapy versus racemic propranolol in the treatment of high-risk IH, to define the optimal dosage regimen and fill the gap in clinical evidence. Second, the conduct of stereoselective pharmacokinetic/pharmacodynamic (PK/PD) studies in the infant population. These studies should clarify the metabolic characteristics and concentration-effect relationship of the R- and S-enantiomers in infants, to provide a scientific basis for individualized drug administration. Third, the in-depth mechanistic research and formulation development. Structural biology studies should be performed to verify the direct binding of R-(+)-propranolol to SOX18, and pediatric-appropriate oral formulations of R-(+)-propranolol, as well as topical formulations for superficial IH, should be developed to further improve the safety and treatment adherence of IH therapy.

## 5 Discussion

**Figure 1:**
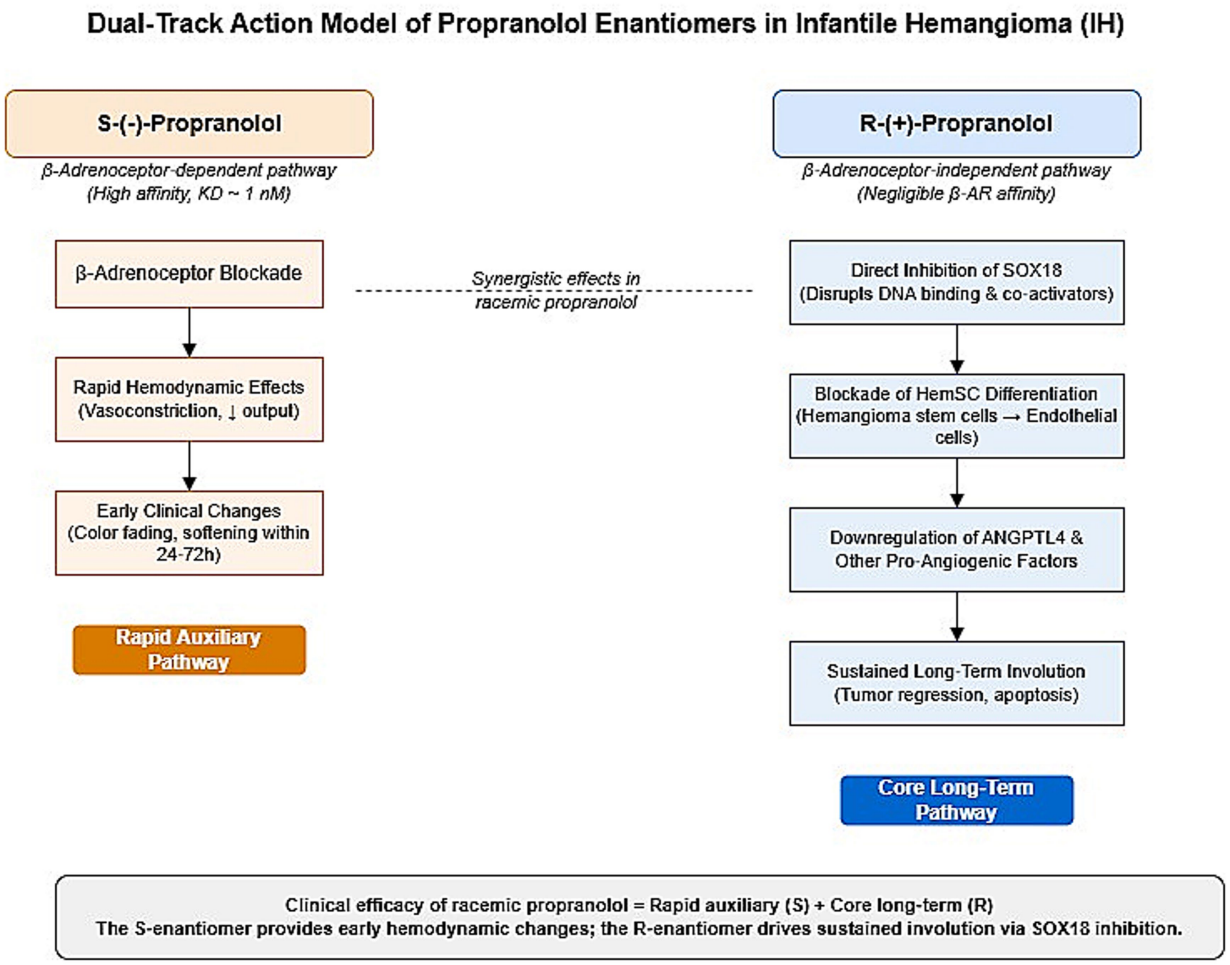
Dual-track action model. Left: *β*-adrenoceptor-dependent rapid auxiliary pathway of S-propranolol. Right: β-adrenoceptor-independent core long-term pathway of R-propranolol via SOX18 inhibition. We propose this dual-track model as a hypothesis-generating framework that reconciles current preclinical observations, rather than a conclusively proven clinical mechanism. Direct clinical validation is required to confirm the differential roles of the two enantiomers in IH involution.

In this review, the authors systematically integrated the full evidence chain of the *β*-adrenoceptor-independent anti-IH activity of R-(+)-propranolol, and proposed an updated dual-track synergistic action model of propranolol in IH treatment, which systematically revises the traditional single *β*-adrenoceptor-dependent mechanism. This model clarifies that the therapeutic effect of propranolol is jointly mediated by two independent and synergistic pathways: the rapid auxiliary pathway mediated by S-(−)-propranolol via *β*-adrenoceptor blockade, which explains the early hemodynamic improvement of lesions, and the core long-term pathway mediated by R-(+)-propranolol via β-adrenoceptor independence, which is proposed to drive the substantial and sustained involution of IH lesions. This model provides a plausible resolution to the contradictions between traditional mechanistic hypotheses and clinical phenomena, and provides a brand-new theoretical framework for mechanistic research and treatment optimization of IH (Figure 1).

### 5.1 Balanced analysis of core academic controversies in the field

Deepening research on R-(+)-propranolol has identified three core academic controversies that underlie the disconnect between preclinical research and clinical practice, and act as key bottlenecks restricting the clinical translation of R-(+)-propranolol.

The first two controversies focus on the mechanistic basis of R-(+)-propranolol’s effects. The first centers on the relative weight of *β*-adrenoceptor-dependent and independent pathways: one school holds that β-blockade only drives early hemodynamic changes in lesions, while long-term involution relies on R-(+)-propranolol’s non-receptor activity. Key evidence supporting this view includes: in a human xenograft model, R-(+)-propranolol was significantly more effective than S-(−)-propranolol in inhibiting vessel formation (11); furthermore, R-atenolol (also devoid of beta blocker activity) showed anti-IH efficacy comparable to R-propranolol (11); and the specific SOX18 inhibitor Sm4 fully phenocopied the anti-angiogenic effect of R-propranolol (16). The opposing view deems *β*-blockade the core mechanism, citing high β-adrenoceptor expression in IH lesions, the reversibility of propranolol’s effect by β-agonists (25), and non-inferior efficacy of the selective β1-blocker atenolol in large randomized trials (15). Our neutral view is that β-blockade is indispensable for early therapeutic response, while long-term curative effects are predominantly driven by R-(+)-propranolol’s non-receptor activity.

The second controversy concerns whether SOX18 is a direct and specific target of R-(+)-propranolol: supporting data from molecular and cellular experiments confirm R-(+)-propranolol binds the SOX18 HMG domain to block its transcriptional activity (11, 16), while critics note the lack of direct structural biological evidence to confirm physical binding (7), and potential off-target inhibition of other homologous SOX family members. The authors acknowledge that current evidence is limited to cellular experiments, with target specificity and direct binding requiring further validation.

The third controversy relates to the non-inferiority of R-(+)-propranolol monotherapy versus racemic propranolol. Proponents highlight that monotherapy retains core anti-IH efficacy while eliminating *β*-blockade-related adverse events (23), offering significant safety benefits for infants and high clinical translation value; opponents argue that β-blockade is a key component of propranolol’s efficacy, with monotherapy potentially inferior for rapidly progressive high-risk IH (6), alongside unknown long-term infant safety and the 15 + years of real-world clinical data supporting racemic propranolol (4, 24). The authors hold that while preclinical data validate R-(+)-propranolol’s anti-IH activity, its clinical value cannot be overstated prior to clinical trial completion, with non-inferiority requiring confirmation via rigorous multicenter randomized controlled trials (5).

While the S-enantiomer is associated with β-blockade-related adverse events, its role in early hemodynamic changes (e.g., vasoconstriction and lesion color fading) represents an auxiliary therapeutic component that should not be entirely discounted. The potential synergistic interactions between the R- and S-enantiomers warrant further investigation.

### 5.2 Critical appraisal of preclinical model relevance

When appraising preclinical evidence, it is important to consider the relevance of different models to human IH. The human primary HemSC-based xenograft model used by Seebauer et al. (11) is currently considered the gold standard, as it closely recapitulates human IH pathophysiology. In contrast, the bEnd.3 cell line used by Sasaki et al. (20) is a murine endothelioma cell line immortalized by retroviral expression; it is not derived from human IH tissue, and its proliferation and vasculogenesis properties may differ substantially from those of human IH. Therefore, the findings of that study should be regarded as preliminary, and we have accordingly assigned lower weight to this evidence in the present review. Future validation studies should prioritize human-derived or patient-derived xenograft models.

### 5.3 Causal link between molecular mechanism and functional effects of R-(+)-propranolol

The relationship between R-(+)-propranolol’s action on SOX18 and its anti-angiogenic effect can be understood at several levels. Biochemical assays show that R-(+)-propranolol directly binds the HMG domain of SOX18, suppresses its transcriptional activity, and disrupts SOX18 dimerization and SOX18-RBPJ interaction (16). This molecular interference leads, at the transcriptional level, to downregulation of mevalonate pathway enzymes (HMGCR, HMGCS1, MVK) and NOTCH1 (17). The mevalonate pathway is a key downstream axis regulated by SOX18, and the fact that statins (HMGCR inhibitors) phenocopy R-(+)-propranolol’s effects further supports the central role of this axis in IH vasculogenesis (17). At the cellular level, these transcriptional changes translate into impaired HemSC-to-endothelial differentiation and reduced tube formation (11). In animal models, the final outcome is significantly decreased human vessel density and reduced xenograft volume (11, 17). SOX18 knockdown or the specific inhibitor Sm4 recapitulates the above effects (11, 16, 17).

In line with this causal chain, Holm et al. further demonstrated that R-(+)-propranolol downregulates the expression of multiple key enzymes in the mevalonate pathway (MVP), including HMGCR, HMGCS1, and MVK, and that statins (simvastatin, atorvastatin) suppress HemSC-to-endothelial differentiation *in vitro* and reduce human vessel formation in a xenograft model, phenocopying the effect of R-(+)-propranolol (17). These findings provide independent validation of the SOX18-MVP axis as a key downstream effector pathway. Thus, the *β*-adrenoceptor-independent anti-IH effect of R-(+)-propranolol is not a collection of isolated phenomena but a hierarchical biological process driven by a single molecular target.

**Figure 2:**
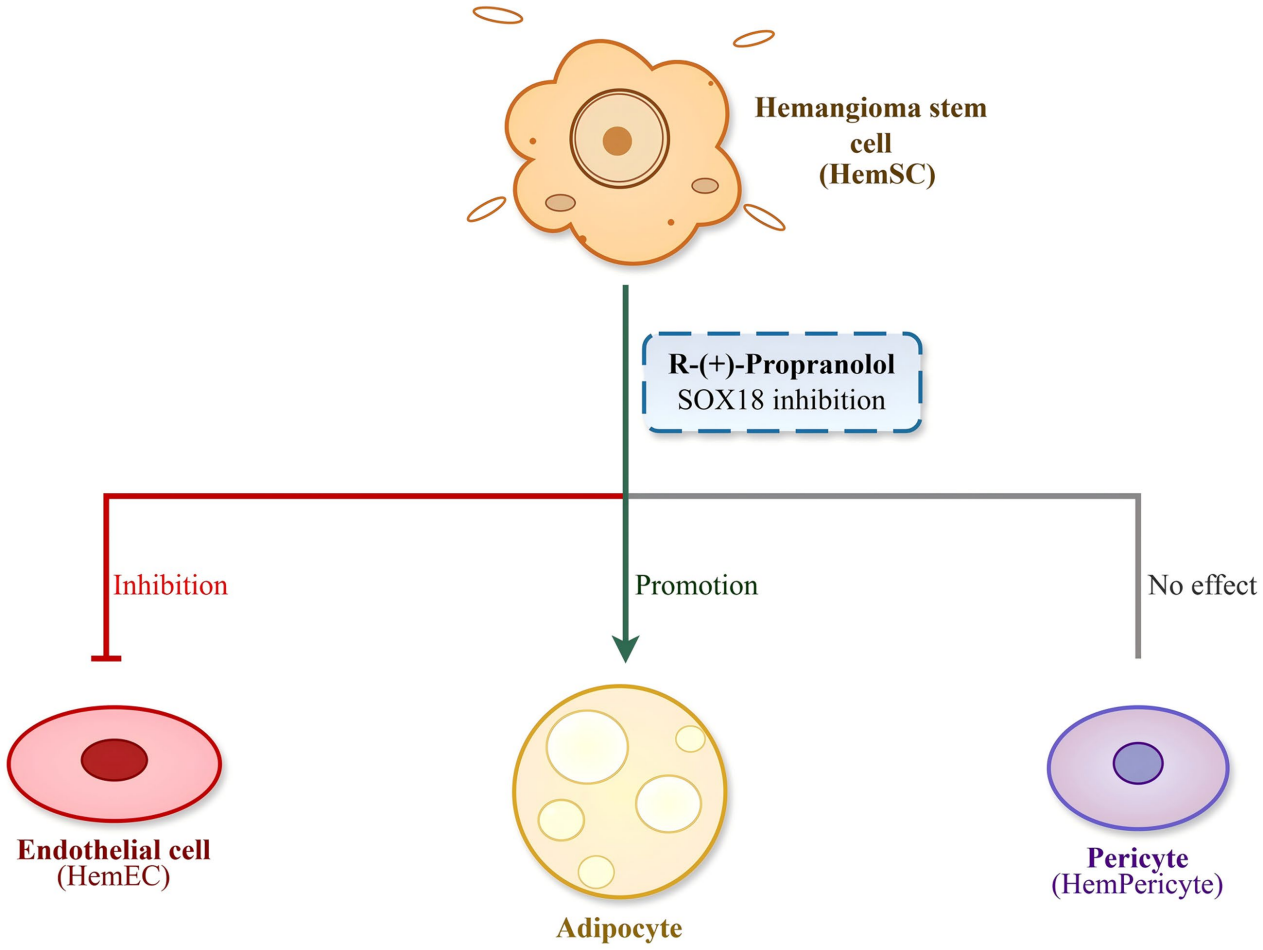
Hypothetical model of R-(+)-propranolol action on HemSC lineage commitment. Hemangioma stem cells (HemSCs) differentiate into three lineages (solid gray arrows): Endothelial cells (HemECs), adipocytes, and pericytes (HemPericytes). R-(+)-propranolol, through SOX18 inhibition, is proposed to block endothelial differentiation, promote adipogenic differentiation, and exert no significant effect on pericyte differentiation.

In addition to its effects on endothelial and adipogenic differentiation, R-(+)-propranolol selectively modulates HemSC fate without affecting pericyte differentiation. Seebauer et al. demonstrated that R-(+)-propranolol does not inhibit the differentiation of HemSCs into pericytes (HemPericytes) either *in vitro* (co-culture with endothelial cells) or *in vivo* (xenograft model), as evidenced by unaltered expression of pericyte markers (calponin, PDGFR*β*, αSMA) and preserved perivascular coverage (11). In contrast, racemic propranolol has been shown to increase pericyte contractility (26). More recently, Chen et al. (27) provided preclinical evidence that R-(+)-propranolol does not impact HemSC-to-pericyte differentiation *in vivo*. Thus, R-(+)-propranolol exhibits a selective regulatory profile on HemSC lineage commitment: it inhibits endothelial differentiation, promotes adipogenesis, while leaving pericyte differentiation intact. This selectivity may preserve vessel stability during involution and could represent a safety advantage over non-selective *β*-blockers (Figure 2).

Table 2 provides a systematic comparison of S-(−)- and R-(+)-propranolol across nine parameters, including primary target, mechanism of action, enantioselectivity, onset of action, clinical observation, dose dependency, safety, *in vivo* efficacy, and clinical translation potential.

**Table 2:** Provides a systematic comparison of S-(-)- and R-(+)-propranolol across nine parameters.

Parameter	S-(−)-propranolol	R-(+)-propranolol
Primary molecular target	β₁- and β₂-adrenergic receptors (GPCRs)	Transcription factor SOX18 (HMG domain)
Mechanism of action	Competitive antagonism of β-ARs → ↓ cAMP → ↓ PKA signaling	Direct binding to SOX18 HMG domain → inhibition of transcriptional activity → disruption of SOX18 homodimerization and SOX18-RBPJ interaction
Enantioselectivity (β-AR affinity)	High affinity (Kd ≈ 1–10 nM)	Negligible β-AR affinity (≈100-fold higher concentration needed for weak β-blockade)
Onset of pharmacological effect	Rapid (hours to days): early vasoconstriction and lesion softening	Slower (days to weeks): requires sustained administration to inhibit vasculogenesis
Clinical observation in IH	Early color change (red to purple) and softening	Progressive, long-term involution with reduced vessel density
Dose–response relationship	Saturable at low concentrations (<0.1 μM); no additional β-blockade with higher doses	Concentration-dependent inhibition of SOX18 activity (broad range 1–20 μM); efficacy increases with dose
Safety profile (known/predicted)	Bradycardia, hypotension, bronchospasm, hypoglycemia, sleep disturbances (crosses blood–brain barrier)	No β-AR-mediated adverse effects (predicted); no clinical safety data available
*In vivo* efficacy (IH xenograft models)	Partial inhibition of human vessel formation (weaker than R-enantiomer)	Potent, dose-dependent inhibition of HemSC-derived vessel formation
Clinical translation potential	Already used as part of racemic mixture; adverse effects limit utility	Promising as a safer monotherapy, avoiding β-blockade-related side effects

β-AR, β-adrenoceptor; cAMP, cyclic adenosine monophosphate; PKA, protein kinase A; HMG, high mobility group; RBPJ, recombination signal binding protein for immunoglobulin kappa J region; IH, infantile hemangioma; HemSC, hemangioma-derived stem cell.

“~100-fold higher” refers to β-receptor blocking potency (9, 10).

Data in this table are compiled from Refs. (6, 8–11, 15–17, 20, 23, 24).

### 5.4 Limitations of this review

This review has the following limitations that need to be clarified: First, this review focuses on the *β*-adrenoceptor-independent mechanism of R-(+)-propranolol, and does not carry out a comprehensive and in-depth elaboration of the traditional β-adrenoceptor-dependent mechanism, which may lead to incomplete coverage of the full mechanism of propranolol. Second, most of the included studies are preclinical basic studies, and there is a lack of clinical research data, which leads to certain limitations in the clinical translation value of the conclusions. Third, this review only includes Chinese and English literature, and may miss relevant research published in other languages.

### 5.5 Clinical translation value and prospect of R-(+)-propranolol

The discovery of the β-adrenoceptor-independent activity of R-(+)-propranolol has opened up a new direction for the precision treatment of IH. At present, the clinical treatment of IH still faces two core pain points: first, the adverse events related to β-adrenoceptor blockade of racemic propranolol lead to strict treatment monitoring requirements and high clinical threshold; second, about 10% of children have poor response to propranolol treatment, and there is a lack of individualized treatment plans. The development of R-(+)-propranolol monotherapy is expected to solve these two pain points: on the one hand, it could potentially avoid the cardiovascular and respiratory adverse events caused by *β*-adrenoceptor blockade, theoretically reduce the monitoring requirements of treatment, and potentially improve the safety of medication in infants; on the other hand, it can provide a new treatment option for children with poor response to racemic propranolol.

For patients who respond poorly to racemic propranolol, the potential benefit of R-(+)-propranolol monotherapy may be twofold. First, the dose-limiting adverse effects caused by the S-enantiomer in the racemic mixture often prevent dose escalation, thereby limiting the achievable concentration of the R-enantiomer. Second, individual variations in SOX18 pathway activity may influence therapeutic response; R-monotherapy could offer a more targeted and optimized intervention for these patients.

In addition, the research paradigm of R-(+)-propranolol also provides a classic reference for the “old drug new use” of chiral drugs. For most chiral drugs in clinical use, the pharmacodynamic differences between enantiomers are often ignored, and the development of single-enantiomer preparations based on mechanism research can not only improve the therapeutic effect, but also reduce adverse reactions, which is an important direction for the innovation of clinical drugs in the future.

## 6 Conclusion

This review systematically summarizes the available core evidence and provides preclinical evidence that R-(+)-propranolol exerts a significant, *β*-adrenoceptor-independent anti-IH activity. Its proposed core mechanism of action is the specific inhibition of the transcriptional activity of the transcription factor SOX18, which blocks the differentiation and proliferation of hemangioma endothelial cells at the source, while exerting synergistic anti-angiogenic effects via the regulation of molecules including ANGPTL4. The therapeutic efficacy of propranolol in IH is jointly mediated by the “rapid auxiliary pathway” dependent on β-adrenoceptor blockade mediated by the S-enantiomer, and the “core long-term pathway” independent of β-adrenoceptors mediated by the R-enantiomer, among which the R-enantiomer is the predominant active component in experimental models that may contribute to the long-term involution of lesions.

This discovery has reshaped the understanding of the mechanism of action of propranolol, this classic pharmaceutical agent, and opened up a new direction for the precision therapy of IH. The core mission in the field at present is to verify the efficacy and safety of R-(+)-propranolol monotherapy through rigorous clinical trials, to promote its translation from basic research to clinical practice, and to provide a safer and more precise treatment option for millions of children with IH worldwide.
